# A commentary on ‘Comparative investigation of neoadjuvant immunotherapy versus adjuvant immunotherapy in perioperative patients with cancer: a global-scale, cross-sectional, large-sample informatics study’

**DOI:** 10.1097/JS9.0000000000001712

**Published:** 2024-05-29

**Authors:** Jia Yang, Zhongqi Wang, Haibin Deng

**Affiliations:** Department of Oncology, Longhua Hospital affiliated to Shanghai University of Traditional Chinese Medicine, Shanghai, People’s Republic of China

*Dear Editor,*

The field of immunotherapy has significantly revolutionized cancer treatment, providing new avenues for enhancing patient outcomes during the perioperative period. Perioperative immunotherapy has also emerged as a new hotspot in clinical research^1^. The recent study by Guo *et al*.^2^, published in the *International Journal of Surgery*, offers a comprehensive global, cross-sectional, large-sample informatics analysis that compares neoadjuvant immunotherapy with adjuvant immunotherapy in patients with perioperative cancer. This study is particularly timely, as their results showed that the global focus of immunotherapy shifted from adjuvant therapy to neoadjuvant therapy following 2020, with the efficacy and safety of neoadjuvant immunotherapy becoming a burgeoning area. Notably, the research predicts key yet underexplored research directions, such as non-small cell lung cancer, immune checkpoint inhibitors, pathological complete response, interleukin-2, melanoma, and dendritic cells. This foresight could guide future research to fill existing gaps in our understanding of cancer immunotherapy.

Neoadjuvant and adjuvant immunotherapies have succeeded remarkably in clinical practice, outperforming traditional chemotherapy. Neoadjuvant immunotherapy, administered before surgical intervention, leverages intact tumor antigens and a lymphatic tumor microenvironment to enhance immune efficacy, manage micrometastases, and reduce recurrence risks. In contrast, postoperative adjuvant immunotherapy blocks immune checkpoint expression, such as PD-1/CTLA-4, thus restoring T cell anti-tumor activity and facilitating the capture and killing of cancer cells^3^. However, research exploring the differences in the mechanisms and therapeutic effects between the two therapies remains scarce, underlining an urgent need for enhanced treatment strategies to improve patient prognoses.

Guo *et al*. employed unsupervised hierarchical clustering, time-series analysis, the Walktrap algorithm, and other informatic methods to provide a macroscopic perspective of the current landscape of cancer immunotherapy, highlighting potential future research directions. Despite these advanced methods, the study primarily relies on bibliometric analysis based on literature data, lacking a direct comparison or validation of the exact differences in patient prognosis and clinical efficacy between neoadjuvant and adjuvant immunotherapy. This limitation underscores the necessity for head-to-head prospective phase III clinical trials^4^. Second, although the authors strictly defined the search terms for neoadjuvant and adjuvant immunotherapy, the inclusion criteria did not differentiate among various immunotherapy modalities, such as monotherapy, dual immunotherapy, immunotherapy combined with radiotherapy or chemotherapy, or continuation of adjuvant immunotherapy after neoadjuvant therapy, each of which may lead to different immune responses and toxicities^5^. Further in-depth discussions are needed regarding the various clinical factors associated with different cancer types, tumor stages, pathological types, treatment modalities, biomarkers, and predominant populations. Finally, the study’s methodology could be refined by diversifying the literature sources beyond the Web of Science database to mitigate bias, which the authors recognize and suggest that future studies could broaden database sources.

We are deeply grateful to the authors for making significant contributions to the field of perioperative cancer treatment and also for setting a foundation for future research on optimizing medical resources. Nonetheless, in light of these findings and methodological considerations, it is imperative to conduct more head-to-head prospective clinical trials with longer follow-up periods to validate the advances in immunotherapy. Moreover, further in-depth and detailed exploration is needed in areas such as stratified analysis based on different subgroups, multiple clinical factors, immunotherapy models, screening of advantageous populations, and constructing predictive models.

## Ethical approval

Not applicable.

## Consent

Not applicable.

## Source of funding

This study was supported by the National Natural Science Foundation of China (No. 82274285), Three-year action plan for strengthening the construction of the public health system in Shanghai (GWVI-11.2-YQ17), Clinical Research Plan of SHDC (No. SHDC2020CR4050), and Pudong New Area-High-Level Research-Oriented Traditional Chinese Medicine Hospital Construction (YC-2023-0901).

## Author contribution

J.Y.: conceptualization and writing original draft; Z.W. and H.D.: review and editing.

## Conflicts of interest disclosure

There are no conflicts of interest.

## Research registration unique identifying number (UIN)

Not applicable.

## Guarantor

Jia Yang and Haibin Deng.

## Data availability statement

This manuscript is a comment on a published study, ‘Comparative investigation of neoadjuvant immunotherapy versus adjuvant immunotherapy in perioperative patients with cancer: a global-scale, cross-sectional, large-sample informatics study’, and the data are publicly available.

## Provenance and peer review

Not commissioned, externally peer-reviewed.
